# Identification of Age-Specific and Common Key Regulatory Mechanisms Governing Eggshell Strength in Chicken Using Random Forests

**DOI:** 10.3390/genes11040464

**Published:** 2020-04-24

**Authors:** Faisal Ramzan, Selina Klees, Armin Otto Schmitt, David Cavero, Mehmet Gültas

**Affiliations:** 1 Breeding Informatics Group, Department of Animal Sciences, Georg-August University, Margarethe von Wrangell-Weg 7, 37075 Göttingen, Germany; faisal.ramzan@stud.uni-goettingen.de (F.R.); selina.klees@uni-goettingen.de (S.K.); armin.schmitt@uni-goettingen.de (A.O.S.); 2 Department of Animal Breeding and Genetics, University of Agriculture Faisalabad, 38000 Faisalabad, Pakistan; 3 Center for Integrated Breeding Research (CiBreed), Albrecht-Thaer-Weg 3, Georg-August University, 37075 Göttingen, Germany; 4 H&N International, 27472 Cuxhaven, Germany; cavero@ltz.de

**Keywords:** eggshell strength, chicken, Random Forests, feature selection, master regulators, over-represented pathways

## Abstract

In today’s chicken egg industry, maintaining the strength of eggshells in longer laying cycles is pivotal for improving the persistency of egg laying. Eggshell development and mineralization underlie a complex regulatory interplay of various proteins and signaling cascades involving multiple organ systems. Understanding the regulatory mechanisms influencing this dynamic trait over time is imperative, yet scarce. To investigate the temporal changes in the signaling cascades, we considered eggshell strength at two different time points during the egg production cycle and studied the genotype–phenotype associations by employing the Random Forests algorithm on chicken genotypic data. For the analysis of corresponding genes, we adopted a well established systems biology approach to delineate gene regulatory pathways and master regulators underlying this important trait. Our results indicate that, while some of the master regulators (*Slc22a1* and *Sox11*) and pathways are common at different laying stages of chicken, others (e.g., *Scn11a*, *St8sia2*, or the TGF-β pathway) represent age-specific functions. Overall, our results provide: (i) significant insights into age-specific and common molecular mechanisms underlying the regulation of eggshell strength; and (ii) new breeding targets to improve the eggshell quality during the later stages of the chicken production cycle.

## 1. Introduction

Today’s poultry industry is highly invested in the development of chicken capable of producing more eggs in longer laying cycles [1]. This production goal, however, must go hand in hand with improvement in sustainability of egg quality, especially eggshell strength (ESS), during the whole laying period [1,2]. The calcified eggshells not only provide protection against physical damage but also play a crucial role for the development of the embryo by allowing gaseous exchange, abating moisture loss, and supplying calcium for the embryo bone development [3]. Multiple molecular actors involved in the homeostasis and transportation of minerals, especially calcium, the main constituent of the eggshell, have been identified [4,5]. More than 500 eggshell matrix proteins have also been reported [6,7] implicating a plethora of genes that knit together the complex protein scaffold and the mineral phase of the eggshell [5,8]. However, most of these discoveries provide only the genes expressed in a certain segment of the chicken oviduct, the principal organ for egg development, and consequently the overall mechanisms of eggshell development remain illusive. Moreover, similar to other economically important traits, ESS remains relevant throughout the productive life and commonly deteriorates with the age of the chicken [9]. This decline in the eggshell quality remains one of the major reasons for replacing commercial flocks [1]. Hence, understanding the genetic basis of ESS at different laying stages is very important for breeders if they are to extend the laying cycle of chicken. Therefore, an analysis of this trait at different time points during the life of the bird can better delineate its genetics and its molecular mechanisms involved in this dynamic behavior [10]. This knowledge can then be utilized to design breeding strategies to improve the eggshell quality during the later stages of the chicken production cycle.

Until now, a variety of association studies have been conducted to decipher the genetic architecture of quantitative traits such as ESS, which led to the identification of a valuable repertoire of genes controlling a range of traits (see the reviews [11,12,13]). Finding loci associated with a trait through genome wide association studies (GWAS) is commonly based on single-SNP based models that test each SNP for its association with the phenotype, ignoring its dependency on the neighboring SNPs. This statistical design of GWAS seems quite straightforward, yet entails several challenges including those of population stratification, relationships among the samples, multiple hypothesis testing, and overestimation of SNP effects, among others, as pointed out in previous studies [14,15,16,17]. Many approaches such as different multiple testing correction methods and linear mixed models have been proposed to overcome these challenges [15,18,19]. However, the most devastating challenge of GWAS still persisting is the lack of power to detect the loci having medium to small effect sizes [20]. This inability of GWAS to explain a major proportion of the heritability has been under intensive discussion.

To overcome these limitations of GWAS, application of Bayesian frameworks as well as machine learning algorithms have gained importance in the last decade [21,22,23,24,25]. Their comparative performance has been evaluated for a variety of traits with different genetic architectures (see the reviews [13,26,27]). Nevertheless, multiple studies have revealed that machine learning algorithms surpass currently available well-known GWAS approaches in identifying genes having small effects on the phenotype [28,29,30]. In particular, Brieuc et al. pointed out the efficiency of Random Forests (RF) models for analyzing a large number of loci simultaneously and identifying promising associations [28]. Inspired by Brieuc et al.’s study, we applied the RF algorithm to assess the importance of SNPs that could provide a clue of their essential roles for ESS and to characterize the differences observed in this trait at different time points. For the analysis of the corresponding genes of these SNPs, we adopted a well established systems biology approach and identified age-specific and common key regulatory pathways and master regulators. These findings could: (i) enhance our understanding of the regulatory mechanisms underlying eggshell strength; and (ii) provide novel targets and hypotheses for future breeding strategies. To the best of our knowledge, it is the first study in this field which mainly focuses on the importance of the age-specific and common key regulatory mechanisms in chicken to reveal the genetic programs influencing the eggshell strength.

## 2. Materials and Methods

**Figure 1:**
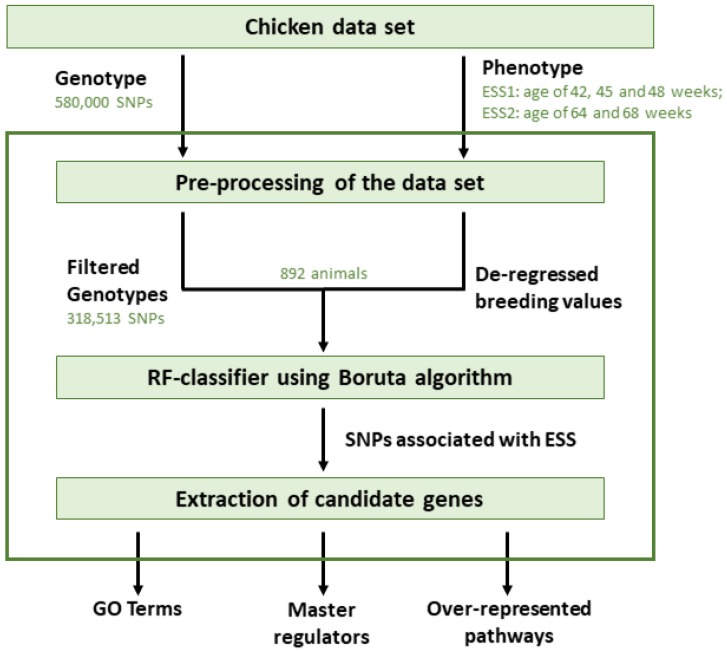
Flowchart of the analysis applied in this study (ESS, Eggshell strength).

In this section, we describe the chicken dataset analyzed and the methods applied. Our analysis follows the structure of Figure 1.

### 2.1. Chicken Dataset

To explore the genomic background of the changes that incur to the eggshell strength during the life of laying birds, we analyzed a genotype dataset that has previously been used to investigate the accuracy of imputation as well as the prediction of genomic breeding values in chicken [31,32,33]. The dataset consists of a purebred commercial brown layer line with 892 animals and 580,000 SNPs generated using Affymetrix Axiom Chicken Genotyping Array. The genotypic data do not contain mitochondrial SNPs. The corresponding phenotypic data consist of eggshell strength (ESS) measured (as the force in Newton that was required to break the eggshell) for each bird at two distinct stages of its production cycle. These two stages were then regarded as Time Point 1 and Time Point 2, respectively. The first time point for ESS was recorded at the ages of 42, 45, and 48 weeks and the second time point was recorded at the ages of 64 and 68 weeks. Averages of the recorded breaking strengths at Time Point 1 (ESS1) and Time Point 2 (ESS2) were used as phenotypes in the further analysis. Extensive pedigree data, consisting of, in total, 40,545 individuals from six generations, were available on these birds which were included in an animal model for breeding values estimation of the birds. These breeding values were then de-regressed following Garrick et al. [34] to obtain the pseudo-phenotypes that were used in the further analysis. To ensure genotype quality, we filtered the genotyped data and removed the SNPs: (i) that were unassigned to any chromosome or present on the sex chromosomes; (ii) with a minor allele frequency < 0.01; (iii) with a genotyping call rate ≤ 97%; (iv) significantly deviating from Hardy–Weinberg equilibrium (*p*-value $<1\times 10^{-6}$); and (v) for animals having a SNP call rate smaller than 95%. Finally, after filtering, we used 892 animals and 318,513 SNPs for our analyses.

### 2.2. Association Analysis Using Random Forests

To identify SNPs potentially associated with eggshell strength, we used the concept of the Random Forests (RF) algorithm to estimate the relative importance of each SNP (attribute) regarding its involvement in the prediction of response variables (de-regressed breeding values). For this purpose, we employed the Boruta algorithm in our study [35], which is a specially developed powerful wrapper for the RF based feature selection approach. The main principle of the Boruta algorithm is based on the extension of the attributes by adding random attributes to the dataset which are called *shadow attributes* and created by shuffling the original values of each attribute (in our case SNPs) in the dataset. The enlargement of the attributes results in apposition of the randomness to the dataset, which leads to the reduction of the bias of hidden (false) signals arising from random fluctuations or correlations in the dataset [35,36,37]. To this end, a RF classifier is applied to the extended dataset, and SNPs are systematically and iteratively removed whose importance are significantly smaller than those of the *shadow attributes*. By repeating the process of *shadow attributes* generation and RF algorithm application, importance is assigned to all SNPs. As a result, the Boruta algorithm provides a ranked list of SNPs with a decision of whether the importance of a SNP is confirmed, rejected, or tentative. It is important to note that a similar idea to the Boruta algorithm is manually implemented in [22] to assess the importance of SNPs.

### 2.3. Gene Set Analysis

We extracted the genes corresponding to the SNPs identified by the Boruta algorithm from Ensembl using BioMart [38] (R-script given in File S1). Furthermore, we performed a gene set analysis regarding their molecular functions to obtain functional annotations of these genes.

### 2.4. Identification of Master Regulators and Over-Represented Pathways

Following our previous studies [39,40], we performed the "upstream analysis” and pathway analysis using the geneXplain platform [41] to gain more insight into the functional relationships of genes. The algorithm of “upstream analysis” workflow was introduced by Koschmann et al. [42] and its main goal is to reveal the underlying key regulators that control the activity of target genes. For this purpose, the underlying algorithm of “upstream analysis” firstly constructs molecular pathway networks and then detects convergence points of these networks, which are called master regulators and are likely to orchestrate the transcriptional regulation of several genes. In our analysis, we used the GeneWays database [43] and ran the standard “upstream analysis” workflow with a maximum radius of 10 steps upstream to identify the top five master regulators of each gene set resulted from the previous step of the analysis.

To discover novel biological functions and to reveal the properties of the genes under study, we performed a pathway enrichment analysis as the second step of our analysis. To this end, we used the TRANSPATH pathway database [44], which is a regularly updated signaling pathway database and contains information about genes, molecules and reactions for the identification of age-specific and common over-represented pathways.

## 3. Results

**Figure 2:**
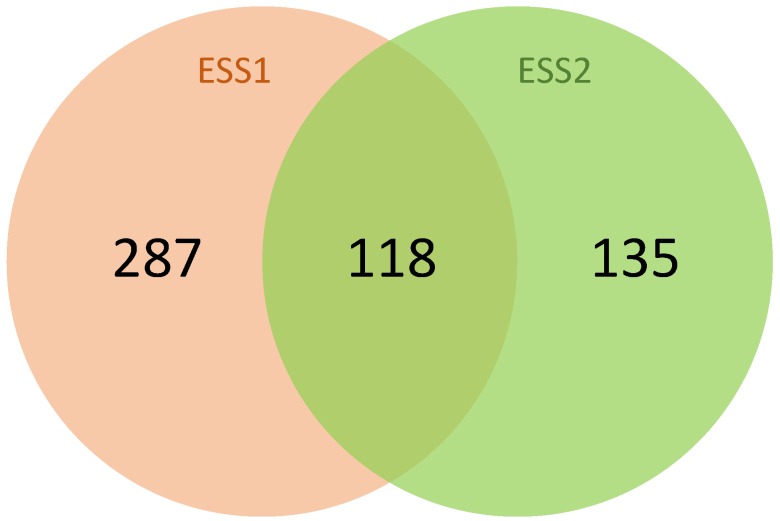
Venn diagram depicting the number of genes associated with eggshell strength at Time Point 1 (ESS1), at Time Point 2 (ESS2), and their overlap.

In this study, we performed the RF approach using the Boruta algorithm to identify the informative SNPs associated with eggshell strength at two time points during the laying cycle of commercial brown layer chicken. For this purpose, the importance of each SNP was separately assessed for its association with the phenotype of interest. To this end, we obtained a list of SNPs for each time point whose importance was confirmed by the Boruta algorithm for the prediction of the phenotype. Analyzing both time points, we identified 3726 SNPs associated with eggshell strength at Time Point 1 (ESS1) and 1815 SNPs associated with eggshell strength at Time Point 2 (ESS2) (the lists of SNPs are given in Table S2). These SNPs were then mapped to the genome and the genes harboring at least one of these SNPs were identified for both traits. In total, we identified 405 genes for ESS1 and 253 genes for ESS2 (the lists of genes and their Gene Ontology (GO) categories are given in Tables S2 and S3, respectively). A closer look at these gene lists reveals that 22 % (118 genes) of them are overlapping (see Figure 2), which depicts the conservation of some of the underlying mechanisms involved in the synthesis of eggshell during different stages of the egg production cycle. Our results also show that a considerably high number of genes that were distinct for the time points highlight the dynamic nature of this trait.

This section is comprised of three parts. First, to gain a deeper insight into these gene sets, we performed a gene set analysis and clustered their functions based on the GO terms. Second, we performed the “upstream analysis” introduced by Koschmann et al. [42] for the identification of specific and common master regulators of both time points. Third, we present the over-represented pathways to further elucidate the mechanisms that control the ESS at different production stages of birds.

### 3.1. Gene Set Analysis

**Figure 3:**
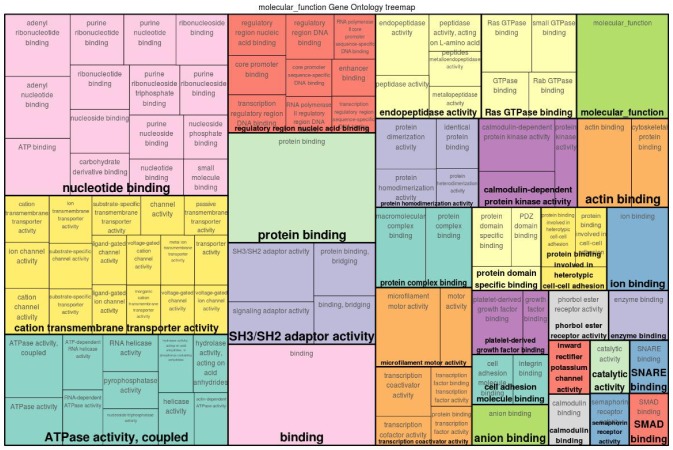
Gene Ontology (GO) treemap for genes associated with eggshell strength at Time Point 1 (ESS1). The boxes are grouped together based on the upper-hierarchy GO-term which is written in bold letters.

**Figure 4:**
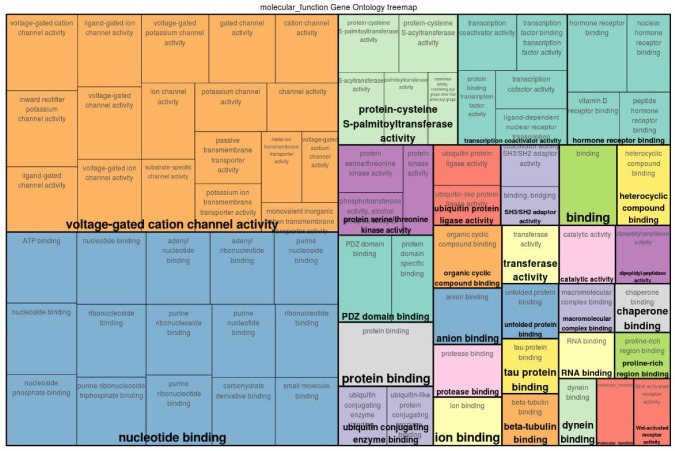
Gene Ontology (GO) treemap for genes associated with eggshell strength at Time Point 2 (ESS2). The boxes are grouped together based on the upper-hierarchy GO-term which is written in bold letters.

The functional classification of both gene sets indicates that there are several GO categories that were common for both time points (see the treemaps depicted in Figure 3 and Figure 4 and the top 15 GO terms in Table 1 and Table 2). In particular, the transportation of cations across membranes was the most salient function for the underlying mechanism of ESS at both time points. In this regard, calcium ions, being the main constituent of the eggshell, are supplied in large amount to the uterine fluid by transepithelial transport. In addition, other cations such as sodium, magnesium, and potassium are exchanged across the uterine endothelium to maintain the cell homeostasis [4,5]. This transmembrane transport remains important during the production cycle to ensure the development of an eggshell. The gene set analysis further reveals that the activities pertaining to ATPase, GTPase, calmodulin binding, calmodulin-dependent protein kinase, and Smad binding were specific for ESS1. Meanwhile, functions related to hormone/vitamin D receptor binding, chaperone binding, and Wnt-activated receptors were more relevant for ESS2.

Among others, the function of ATPase in eggshell formation has been well investigated in previous studies [5,45]. Along with maintaining a pH of the uterine fluid during the eggshell formation, the ATPases also provide the required energy and function as transmembrane transportation channels for ions [46]. The calmodulin binding and calmodulin-dependent protein kinase activity is known to regulate the concentration of calcium in various cells [47] and so does the vitamin D receptor binding [48]. The chaperone binding activity of the genes associated with ESS2 is another distinctive finding of this study. Chaperone proteins have been reported in the uterine fluid where they perform the folding of the eggshell matrix proteins into a rigid scaffold upon which mineralization takes place to produce the fabric of eggshell [5]. These functional classes elucidate the molecular functions that gain more relevance depending on the age of the birds and demonstrate the key functions that remain important throughout the laying cycle of the birds.

### 3.2. Identification of Master Regulators

**Figure 5:**
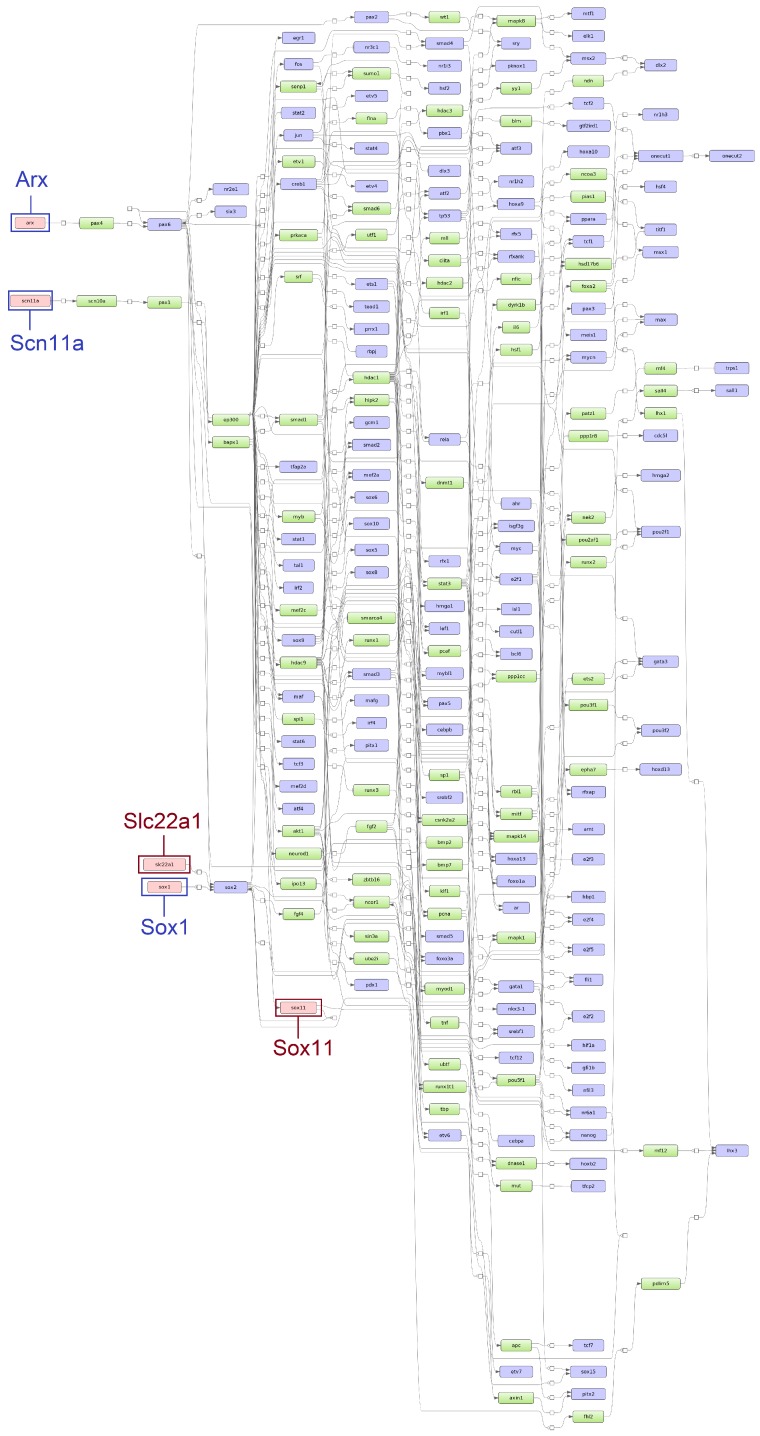
Scheme of gene regulatory pathways revealing the top five master regulators (pink filled boxes) for eggshell strength at Time Point 1 (ESS1) following the “upstream analysis” [42]. The master regulators written in dark blue and surrounded by dark blue boxes (*Arx, Scn11a* and *Sox1*) were identified specifically for ESS1 while master regulators written in dark red and surrounded by dark red boxes (*Slc22a1* and *Sox11*) were identified at both time points (corresponding networks for eggshell strength at Time Point 2 (ESS2) in Figure 6).

**Figure 6:**
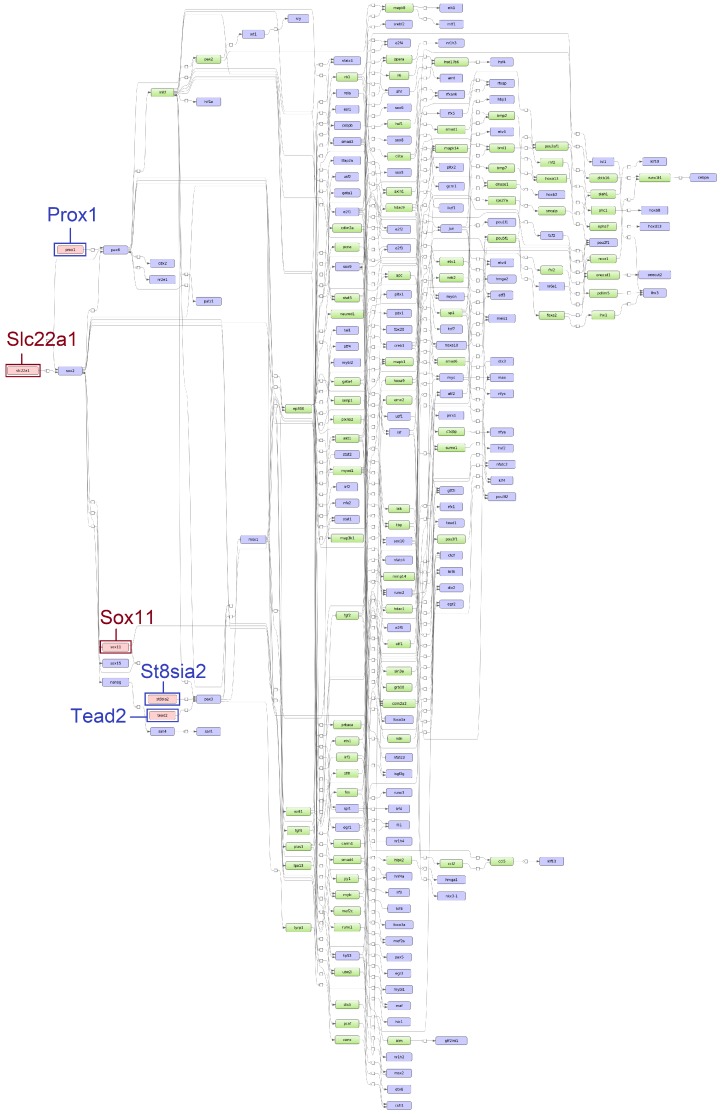
Scheme of gene regulatory pathways revealing the top five master regulators (pink filled boxes) for eggshell strength at Time Point 2 (ESS2) following the “upstream analysis” [42]. The master regulators written in dark blue and surrounded by dark blue boxes (*Prox1, St8sia2* and *Tead2*) were identified specifically for ESS2 while master regulators written in dark red and surrounded by dark red boxes (*Slc22a1* and *Sox11*) were identified at both time points (corresponding networks for eggshell strength at Time Point 1 (ESS1) in Figure 5).

Applying the “upstream analysis” integrated in the geneXplain platform [41], we identified the top five age-specific and common master regulators. While the master regulators *Arx, Sox1,* and *Scn11a* were specifically found for ESS1, the master regulators *St8sia2, Tead2,* and *Prox1* were identified for ESS2. Additionally, *Slc22a1* and *Sox11* were identified for both time points (see Figure 5 and Figure 6).

The ESS1 specific master regulator *Scn11a* is a gene encoding transmembrane sodium channels which control the voltage-gated sodium transport especially in the uterus [49,50], the site of eggshell synthesis in birds. Moreover, the importance of sodium channels in the transportation of inorganic minerals deposited in the eggshell is well established [51]. Interestingly, we found the master regulator *Slc22a1* at both time points. It codes for the protein OCT1, an organic cation transporter for substrates such as putrescine [52], which plays an important role for eggshell thickness [53] and calcium transport in the intestine [54]. Furthermore, many other members of the super-family of transport proteins, *Slc* (solute carrier proteins), are well known to play an essential role in the homeostasis of calcium ions in a variety of tissues [55]. The *Slc* proteins have also been reported to transport magnesium ions during the egg calcification process [5].

Another interesting master regulator, *Sox11*, which encodes a member of the Sox (SRY-related HMG-box) family of transcription factors, was found at both time points. *Sox11* is known to positively regulate the process of osteogenesis (the formation of bone) [56]. This regulator gains relevance given the importance of bone as a labile reservoir of minerals, especially calcium [4]. In birds, the calcium homeostasis is achieved by regulating the metabolism of bone minerals as well as by controlling the absorption and excretion of calcium in the intestine and in kidneys, respectively [57]. Furthermore, the master regulator *Tead2* found for ESS2 is a regulator of osteogenesis [58] and it is also one of the direct downstream target genes of *Sox11*. This might be an indication of different regulatory mechanisms involved in the osteogenesis or bone remodeling during the later stages of the laying cycle [56].

The *St8sia2*, identified as an ESS2 specific master regulator, encodes a membrane protein which catalyzes the metabolism of sialic acid [59], a carbohydrate found in the eggshell membranes [60,61,62]. The eggshell membranes constitute the inner layer of the eggshell and contribute to its strength. They further provide the nucleation sites for the initiation of the shell synthesis [63]. Sialic acid is also part of podocalyxin and secreted phosphoprotein 1 (SPP1), both of which are glycoproteins found in the uterus during eggshell calcification [5,64]. Because of its high negative charge, podocalyxin is presumed to interact with calcium carbonate during the calcification of the eggshell [64]. The master regulator *Prox1* encodes the protein prospero homeobox 1 that has also been reported as part of eggshell membranes [65,66]. However, the *Prox1* gene is mostly implicated in the regulation of the development of a variety of organs including liver, pancreas and kidney [67]. Although the vast majority of the master regulators could be biologically characterized to be crucial for ESS, the importance and role of the two master regulators *Sox1 and Arx* for this trait is currently biologically unconfirmed and could hence provide novel targets for future studies.

### 3.3. Identification of Over-Represented Pathways

**Figure 7:**
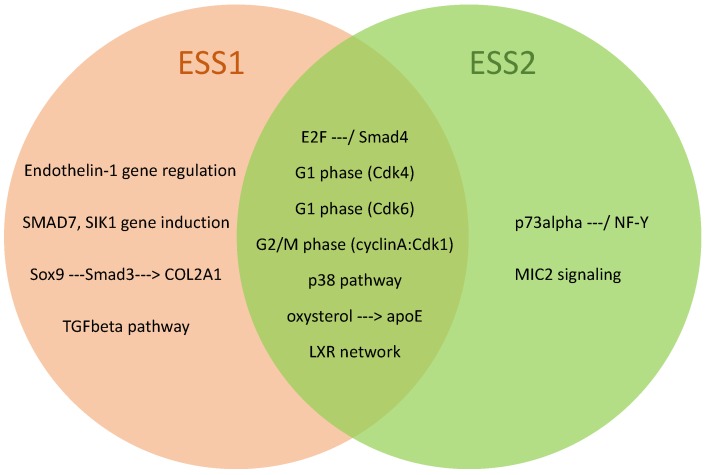
Venn diagram of over-represented pathways (*p* adjusted < 0.001) of eggshell strength at Time Point 1 (ESS1), at Time Point 2 (ESS2), and their overlap. Pathways are based on the TRANSPATH pathway database [44].

To further elucidate and investigate the mechanisms that control the ESS at different time points, we were interested in identifying age-specific and common over-represented pathways. Applying the pathway analysis, we identified eleven and nine significantly over-represented pathways for ESS1 and ESS2, respectively, and seven of these pathways are overlapping for both time points (see Figure 7 and Table 3).

Among the pathways shared by both time points, G1 phase (Cdk4), G1 phase (Cdk6), and G2/M phase (cyclinA:Cdk1) involve different members of the cyclin-dependent kinase (CDK) family which regulate transcription, mRNA processing, and, more importantly, cell cycle [68]. In the context of ESS, these pathways may influence the differentiation efficiency of osteoblasts, osteoclasts, chondrocytes [69], and uterine epithelium cells, all of which are crucial for the supply of calcium ions as well as for bone and calcium homeostasis [70,71]. The p38 pathway is implicated in a variety of cellular responses including those related to proliferation, differentiation and apoptosis [72]. Moreover, the role of this pathway has also been reported in the egg development of *Drosophila melanogaster* [73]. The LXR (liver X receptors) network plays a central role in the transcriptional control of lipid metabolism [74]. This pathway also mediates the concentrations of oxysterols and ApoE (Apolipoprotein E) if activated in response to elevated intra-cellular cholesterol levels [75]. The oxysterols, oxygenated forms of cholesterol, are intermediates in bile acid and steroid hormone biosynthetic pathways [76]. Among other steroid hormones, estrogen is more intimately involved in calcium homeostasis and has also been implicated in the development of osteoporosis [77]. Moreover, other forms of oxysterols are also involved in calcium metabolisms [78] and mesenchymal stem cell differentiation [79]. In addition to the CDKs, the Smad4 proteins, predominantly present in the nucleus of the cell, mediate the cell cycle due to their association with the E2F family of transcription factors [80]. These pathways can be upstream regulated by the transforming growth factor β (TGF-β) [81].

The transforming growth factor-β (TGF-β) signaling pathway can be regarded as the most important pathway enriched for ESS1. This pathway, among its other functions, is well-known for its role in bone homeostasis [82]. Furthermore, some components of this pathway also overlap with other pathways delineated in our analysis. The Sox9 is a transcription factor that regulates the expression of the COL2A1 (collagen type II, alpha 1) gene which contributes to collagen formation [83]. During this process, Smad3, a member of effector molecules in the signaling pathways of the TGF-β ligand superfamily is activated [84]. Another pathway that is based on Smad7, SIK1 gene induction also regulates TGF-β signaling [85]. Owing to this crosstalk with a variety of other pathways, the TGF-β signaling pathway allows the bone to adapt to dynamic environments [82].

The Endothelin-1 gene (ET-1) regulation pathway includes the mechanisms regulating ET-1 gene expression. Among other functions, ET-1 is involved in osteoblast proliferation and differentiation in bone tissue as well as in the ovulation process in the uterus [86]. ET-1 gene regulation is responsive to intracellular calcium and calmodulin [87]. The MIC2 signaling pathway, which was specifically enriched for ESS2, has CD99 as the main cell surface protein and has been implicated in apoptosis, adhesion, differentiation, and protein trafficking possibly by affecting actin cytoskeleton reorganization [88,89]. Another ESS2 specific pathway involves the inactivation of the nuclear factor Y (NF-Y) transcription factor by p73 proteins, a process that represses the promoter of the telomerase catalytic subunit and induces replicative senescence [90,91]. The activity of NF-Y is further linked to the parathyroid hormone, which is the main regulator of calcium and phosphorus homeostasis. Taken together, the pathways show a diversity of complex functional features in chicken in response to age-dependent changes in eggshell formation. Some pathways show a direct relevance for ESS while others seem to be indirectly linked via interactions between pathways and regulators [92,93].

## 4. Discussion

To uncover the associations between genetic variants and phenotypes, genome wide association studies (GWAS) have become the method of choice [12]. Despite their success in identifying a multitude of genes, the prediction performance of single-SNP based GWAS strategies is limited [15,17,94]. Alternatively, multi-marker models including different Bayesian frameworks were introduced for GWAS. In these models, all SNPs are fitted simultaneously as random effects assuming a certain prior distribution of SNP effects [13]. In practice, these SNP effects are unknown and may not even strictly follow a certain distribution [25]. Unlike these traditional statistical models, machine learning methods do not require these prior assumptions about the genetic architecture of traits and have been applied in GWAS in humans [30] as well as in livestock [27,95]. Especially, Romagnoni et al. [30] and Huang et al. [24] showed that machine learning based algorithms provide promising prediction power to assess genotype–phenotype associations. In particular, the Random Forests (RF) algorithm has been successfully applied for this purpose. These articles encouraged us to utilize RF in our study since the application of GWAS to identify genetic variants associated with ESS was futile.

Applying RF, we were able to identify a remarkably high number of genes related to ESS which is in agreement with the findings of Maan et al. [6,7], Mikšík et al. [96,97], and Brionne et al. [5], who pointed out a large number of genes/proteins involved in ESS due to the complexity of this trait. The large difference in the number of genes identified for ESS1 and ESS2 reflects the change in the genetic and environmental components of the phenotypic variance over age, as has been reported before for complex traits [98,99]. The overlap between the genes for both time points (see Figure 2) reflects that certain molecular functions remain relevant to eggshell development during the laying cycle of chicken. Particularly, the similarity of genes responsible for the transportation of ions is in line with the findings of Park et al. [100] and Fan et al. [51] who found that the concentration level of different ions in blood does not change with the age of chicken. Interestingly, a closer look at the biological processes of these traits reveals that, while highly significant GO terms are involved in development for ESS1, the significant biological processes for ESS2 are rather related to different metabolic processes (Table S3). The differences in biological processes at both time points could be associated with the temporal changes in the signaling cascades influencing dynamic behavior of eggshell strength over time.

In line with previous studies [39,42,101,102,103], we applied a systems biology approach and identified master regulators to investigate and unravel the transcriptional regulatory machinery of ESS associated genes. Interestingly, our results show that, similar to the genes, there are common master regulators (*Sox11* and *Slc22a1*) for both time points, which are likely to govern various eggshell related processes during the laying of the birds. In particular, being a member of the *Slc* superfamily which is involved in the transmembrane transport, the *Slc22a1* could be essential to eggshell development. For ESS1, the most promising master regulator *Scn11a* controls sodium transport in the uterus [49,50] to maintain a voltage difference as well as osmolarity across uterine cell membranes to help in the calcium transportation [51]. In ESS2, the master regulator *Tead2* together with the master regulator *Sox11* underline the importance of bone remodeling during the later stages of the production cycle of the chicken.

Another fundamental step of our analysis was the identification of the over-represented pathways. The results of this analysis also reinforce the findings of gene set analysis as well as the identified master regulators. Some of the over-represented pathways were conserved at both time points while others were age-specific. Here, we specifically highlight the well-characterized TGF-β pathway that interacts with most of the identified pathways in our analysis to regulate bone homeostasis and thus might play an important role in ESS [82]. The majority of the remaining pathways, especially those which are common to both time points, were found to be related to the cell cycle. The uterine epithelium and bone are the tissues that actively take part in the development of eggshell, hence the renewal of the cells of both tissues is crucial for the synthesis of a strong eggshell [4]. Furthermore, multiple studies suggest that a declining ability of uterine epithelium cells to transport calcium is the main reason of the age-related deterioration of eggshells [51,100]. In particular, the ESS2 specific p73alpha —/ NF-Y pathway that results in the inactivation of the NF-Y transcription factor by p73 proteins and consequently causes replicative senescence of cells [90] may also point towards the underlying reason for weaker eggshells during the later stages of the production cycle.

Recently, the use of systems biology based approaches to study the traits of economic importance is gaining importance in the field of agriculture [39,102,103,104]. However, one of the major impediments in the use of this knowledge in practical animal breeding is to integrate this large amount of information into traditional genetic evaluation programs [105]. A small group of master regulators such as those identified in our analysis integrated into prediction models can possibly be a remedy and might provide novel breeding targets to improve the economically important trait of ESS. Additionally, the knowledge about the specific pathways such as TGF-β could provide novel hypotheses for further studies.

## 5. Conclusions

In this study, we performed a systematic analysis to investigate the age-specific and common regulatory mechanisms that underlie the dynamic trait eggshell strength in chicken. For this purpose, we applied a RF feature selection algorithm to detect the age-dependent genotype–phenotype associations and then used a well established systems biology approach to highlight the master regulators and regulatory pathways that govern the underlying genetic mechanisms of eggshell development. Our results show that most of the genes identified for the ESS at both time points are in agreement with previous studies. Our findings further indicate that some biological processes related to eggshell development remain conserved across production stages while others are age-specific and thus changing over time. To the best of our knowledge, this is the first study revealing master regulators and over-represented pathways in the context of ESS and our findings should be further utilized to design novel hypothesis for future studies.

## Figures and Tables

**Table 1:** Top 15 Gene Ontology (GO) molecular function terms based on the adjusted *p*-value for the eggshell strength at Time Point 1 (ESS1).

GO Term	GO Title	Number of Genes	Adjusted *p*-Value
GO:0005515	protein binding	281	$5.11\times 10^{-8}$
GO:0005488	binding	331	$1.97\times 10^{-7}$
GO:0043167	ion binding	155	$4.93\times 10^{-3}$
GO:0000146	microfilament motor activity	5	$4.93\times 10^{-3}$
GO:0003779	actin binding	20	$6.9\times 10^{-3}$
GO:0032559	adenyl ribonucleotide binding	49	$1.47\times 10^{-2}$
GO:0030554	adenyl nucleotide binding	49	$1.51\times 10^{-2}$
GO:0044877	macromolecular complex binding	50	$1.54\times 10^{-2}$
GO:0004683	calmodulin-dependent protein kinase activity	5	$1.54\times 10^{-2}$
GO:0005524	ATP binding	47	$2.05\times 10^{-2}$
GO:0042623	ATPase activity, coupled	16	$2.24\times 10^{-2}$
GO:0008092	cytoskeletal protein binding	30	$3.32\times 10^{-2}$
GO:0043168	anion binding	74	$3.93\times 10^{-2}$
GO:0046983	protein dimerization activity	40	$4.15\times 10^{-2}$
GO:0017016	Ras GTPase binding	12	$4.86\times 10^{-2}$

**Table 2:** Top 15 Gene Ontology (GO) molecular function terms based on the adjusted *p*-value for the eggshell strength at Time Point 2 (ESS2).

GO Term	GO Title	Number of Genes	Adjusted *p*-Value
GO:0005515	protein binding	168	$1.30\times 10^{-2}$
GO:0022843	voltage-gated cation channel activity	9	$2.09\times 10^{-2}$
GO:0005242	inward rectifier potassium channel activity	4	$2.10\times 10^{-2}$
GO:0032549	ribonucleoside binding	40	$2.79\times 10^{-2}$
GO:0000166	nucleotide binding	48	$2.79\times 10^{-2}$
GO:0005524	ATP binding	34	$2.79\times 10^{-2}$
GO:0001883	purine nucleoside binding	39	$3.66\times 10^{-2}$
GO:0032559	adenyl ribonucleotide binding	34	$3.66\times 10^{-2}$
GO:0005488	binding	199	$3.66\times 10^{-2}$
GO:0030554	adenyl nucleotide binding	34	$3.66\times 10^{-2}$
GO:0051427	hormone receptor binding	9	$3.66\times 10^{-2}$
GO:0015276	ligand-gated ion channel activity	8	$3.66\times 10^{-2}$
GO:0017076	purine nucleotide binding	39	$3.7\times 10^{-2}$
GO:0022836	gated channel activity	12	$3.83\times 10^{-2}$
GO:0036094	small molecule binding	50	$4.64\times 10^{-2}$

**Table 3:** Significantly over-represented pathways for both time points (*p* adjusted < 0.001) sorted by adjusted *p*-values (based on the smaller one of either ESS1 or ESS2). Pathways are based on the TRANSPATH pathway database [44]. (ESS1/ESS2, eggshell strength at Time Point 1/2).

Pathway Name	Adjusted *p*-Value for ESS1 / ESS2	Over-Represented in
E2F —/ Smad4	$5.05\times 10^{-5}$/$7.99\times 10^{-4}$	ESS1, ESS2
Endothelin-1 gene regulation	$5.05\times 10^{-5}$/ -	ESS1
G2/M phase (cyclin A:Cdk1)	$1.61\times 10^{-4}$/$1.65\times 10^{-4}$	ESS1, ESS2
SMAD7, SIK1 gene induction	$1.61\times 10^{-4}$/ -	ESS1
oxysterol —>apoE	$1.61\times 10^{-4}$/$1.85\times 10^{-4}$	ESS1, ESS2
LXR network	$1.61\times 10^{-4}$/$1.65\times 10^{-4}$	ESS1, ESS2
p73alpha —/ NF-Y	- /$1.65\times 10^{-4}$	ESS2
Sox9 —Smad3—>COL2A1	$5.43\times 10^{-4}$/ -	ESS1
G1 phase (Cdk6)	$7.60\times 10^{-4}$/$7.93\times 10^{-4}$	ESS1, ESS2
G1 phase (Cdk4)	$9.77\times 10^{-4}$/$7.99\times 10^{-4}$	ESS1, ESS2
p38 pathway	$9.77\times 10^{-4}$/$7.99\times 10^{-4}$	ESS1, ESS2
MIC2 signaling	- /$7.99\times 10^{-4}$	ESS2
TGFbeta pathway	$9.53\times 10^{-4}$/ -	ESS1

## Supplementary Materials

The following are available online at https://www.mdpi.com/2073-4425/11/4/464/s1, Script S1: R-script for analysis of SNPs and for the extraction of corresponding genes, Table S1: The list of important SNPs, Table S2: The list of genes, Table S3: Gene Ontology categories
